# Optimizing the Use of Extracorporeal Shock Wave Therapy for CP/CPPS: A Modality-Based Systematic Review and Meta-Analysis Comparing Focused and Radial Devices

**DOI:** 10.3390/jcm15031270

**Published:** 2026-02-05

**Authors:** Min-Jui Wu, Chien-Chang Kao, Ming-Hsin Yang, Chih-Wei Tsao, Chin-Li Chen

**Affiliations:** Division of Urology, Department of Surgery, Tri-Service General Hospital, National Defense Medical Center, Taipei 114, Taiwan; rmjw311@gmail.com (M.-J.W.); weisurger@gmail.com (C.-W.T.)

**Keywords:** chronic prostatitis, chronic pelvic pain syndrome, extracorporeal shock wave therapy, adjunctive therapy

## Abstract

**Background/Objectives:** Chronic prostatitis/chronic pelvic pain syndrome (CP/CPPS) is a multifactorial condition often refractory to standard medical therapy. Low-intensity extracorporeal shock wave therapy (Li-ESWT) is a mechanism-oriented option; however, prior reviews reported substantial heterogeneity, potentially due to pooling different wave-generator modalities despite their distinct physical properties. This study synthesized randomized evidence on Li-ESWT for CP/CPPS and explored a wave-generator modality as a prespecified effect modifier. **Methods:** PubMed, Embase, Web of Science, and the Cochrane Library were searched from January 2015 to 31 October 2025 (date of last search) for randomized controlled trials (INPLASY: 2025120064). Eligible studies compared Li-ESWT (focused, radial, or multifocal) with sham or standard medical therapy (SMT). The primary outcome was total NIH-CPSI at the follow-up closest to 12 weeks. Pooled effects were calculated as weighted mean differences (WMDs) with 95% confidence intervals (CIs). Prespecified subgroup analyses were performed by wave-generator modality and therapy strategy (monotherapy vs add-on to SMT). **Results:** Eight RCTs (n = 455) were included. Li-ESWT significantly improved total NIH-CPSI versus the control (WMD −8.46; 95% CI −12.12 to −4.79; I^2^ = 94.8%). Benefits were observed in both monotherapy and the add-on to SMT trials. By modality, focused devices showed consistent effects (WMD −6.59; I^2^ = 0.0%), whereas radial devices showed an imprecise estimate with extreme heterogeneity (WMD −10.38; 95% CI −21.33 to +0.57; I^2^ = 98.2%). Multifocal devices showed a significant benefit (WMD −10.84; I^2^ = 81.0%). Improvements were mainly driven by pain-domain reduction. **Conclusions:** Li-ESWT provides clinically meaningful symptom relief in CP/CPPS, predominantly through pain reduction. Modality- and strategy-based subgroup findings are exploratory given substantial heterogeneity, limited trials, and no head-to-head comparisons; focused devices showed consistent effects, whereas estimates for radial and multifocal devices warrant cautious interpretation.

## 1. Introduction

Chronic prostatitis/chronic pelvic pain syndrome (CP/CPPS) affects an estimated 2–16% of men worldwide and substantially impairs quality of life [1,2]. It is characterized by persistent pelvic pain and lower urinary tract symptoms (LUTSs) in the absence of an active infection. The underlying mechanisms are multifactorial, potentially involving neurogenic inflammation, pelvic floor dysfunction, and impaired pelvic microcirculation [3]. Consistent with this complexity, standard pharmacologic therapies (e.g., antibiotics and α-blockers) often provide limited or short-lived benefit, and many patients remain symptomatic [4,5]. Therefore, multimodal management is often considered. A similar therapeutic challenge is also encountered in chronic bacterial prostatitis (CBP), in which multimodal strategies—such as adding nutraceuticals to antibiotics—have been explored to improve outcomes [6].

Low-intensity extracorporeal shock wave therapy (Li-ESWT) has emerged as a mechanism-based, nonpharmacologic intervention for CP/CPPS. Proposed biological effects include promotion of angiogenesis, modulation of nociceptive signaling, and facilitation of tissue regeneration [7]. Since the initial randomized controlled trial (RCT) by Zimmermann et al., subsequent studies have suggested that Li-ESWT, used either as monotherapy or as an adjunct to pharmacotherapy, may improve pain and urinary symptoms with a favorable safety profile [8,9,10,11]. Some trials suggest that Li-ESWT added to standard medical therapy may further improve symptoms compared with medical therapy alone, consistent with a multimodal approach [12].

Despite these promising results, optimal treatment protocols remain ill-defined. Previous meta-analyses, including the report by Labetov et al. (2024), have noted substantial heterogeneity, which limits the precision and clinical interpretability of pooled estimates [13]. A key limitation is the frequent pooling of focused and radial shock wave devices. These modalities differ in wave generation, energy distribution, and tissue penetration characteristics; therefore, combining them may obscure modality-specific effects and contribute to between-study heterogeneity [14].

To address these limitations, the present systematic review and meta-analysis evaluates focused and radial Li-ESWT as distinct therapeutic modalities. By stratifying trials based on wave-generator type, we aim to provide modality-specific effect estimates and explore potential sources of heterogeneity, thereby informing future protocol optimization and comparative research in CP/CPPS. Because no head-to-head randomized trials are available, modality-specific findings should be interpreted as exploratory.

## 2. Materials and Methods

### 2.1. Study Design and Search Strategy

This systematic review and meta-analysis of randomized controlled trials (RCTs) was conducted and reported in accordance with the Preferred Reporting Items for Systematic Reviews and Meta-Analyses (PRISMA) 2020 statement [15]. The protocol was prospectively registered on the International Platform of Registered Systematic Review and Meta-analysis Protocols (INPLASY; Registration No. 2025120064).

A comprehensive literature search was performed in PubMed, Embase, Web of Science, and the Cochrane Library. The databases were searched from January 2015 up to 31 October 2025 (search end date) to reflect contemporary clinical practice and current-generation shock wave technologies. Ahead-of-print/ePub articles were eligible if indexed by the search end date; non–peer-reviewed preprints were excluded. We utilized a combination of Medical Subject Headings (MeSH) and free-text terms, combining concepts related to the condition (e.g., “prostatitis,” “chronic pelvic pain syndrome,” “CP/CPPS”) and the intervention (e.g., “extracorporeal shockwave therapy,” “Li-ESWT”). The complete, database-specific search strategies are provided in the Supplementary Materials (Table S1).

Two reviewers (Wu and Chen) independently screened titles/abstracts and assessed full texts for eligibility. Reference lists of relevant systematic reviews and included studies were manually checked to identify additional eligible trials.

### 2.2. Study Eligibility Criteria

Studies were eligible if they met the following criteria: (1) Population: adult men diagnosed with CP/CPPS (NIH Category IIIa or IIIb) without evidence of active infection at enrollment; (2) Intervention: low-intensity extracorporeal shock wave therapy (Li-ESWT) delivered using focused, radial, or multifocal wave generators. Device modality was classified according to the trial report/manufacturer description; multifocal was defined as devices delivering shock waves across multiple focal points or a broadened focal field compared with conventional unifocal focused systems; (3) Comparator: sham (placebo) procedure or standard medical therapy alone (e.g., α-blockers, antibiotics, and/or anti-inflammatory agents); and (4) Study design: randomized controlled trials (RCTs). Trials were eligible whether Li-ESWT was administered as monotherapy or as an adjunct to medical therapy.

We excluded non-randomized or non-comparative studies, retrospective observational studies, animal studies, conference abstracts without full texts, reviews, and editorials. A minimum follow-up of 4 weeks with extractable outcome data was required for inclusion. To address variability in follow-up timing, we additionally conducted a sensitivity analysis restricted to trials reporting follow-ups of ≥12 weeks. Trials that did not report any assessment at or beyond 12 weeks were excluded from this sensitivity analysis. Two reviewers (Wu and Chen) independently assessed full texts for eligibility, with disagreements resolved by consensus. For overlapping patient cohorts, the most recent or most comprehensive dataset was retained.

### 2.3. Data Extraction and Quality Assessment

Data were extracted independently by two reviewers (Wu and Chen) using a predefined standardized form. Extracted variables included study characteristics (author, year, sample size, and design), participant demographics, and Li-ESWT protocol details (device modality, energy flux density, number of sessions, and shocks per session). For clinical outcomes, we extracted final follow-up values (means and standard deviations) at the time point closest to the prespecified primary endpoint (12 weeks). Baseline NIH-CPSI values were also extracted and pooled to assess baseline comparability between groups. For the follow-up–restricted sensitivity analysis (≥12 weeks), we extracted the first available assessment at or beyond 12 weeks (if multiple post-12-week time points were reported). Trials reporting only shorter follow-up (<12 weeks) were excluded from this sensitivity analysis. To avoid double-counting, only one follow-up time point per study was included in each meta-analysis.

Final follow-up values were prioritized. When studies reported only change-from-baseline outcomes, follow-up means were derived by combining baseline and change values, with effect directions harmonized to the NIH-CPSI scale (lower scores indicate improvement). Specifically, follow-up means were calculated as baseline mean ± change mean, according to the change-score definition used in each trial. Follow-up standard deviations were extracted directly when reported. If follow-up SDs were unavailable but baseline and change-score SDs were provided, follow-up SDs were imputed using a correlation-based approach assuming r = 0.5 between baseline and follow-up measures, in accordance with the Cochrane Handbook. When required summary statistics were not explicitly reported, missing SDs were derived from standard errors, confidence intervals, or t-values where feasible. Discrepancies in data extraction were resolved through discussion and consensus.

The methodological quality of included studies was assessed independently by two reviewers using the original Cochrane Collaboration risk-of-bias tool for randomized trials (RoB 1.0). Seven domains were evaluated: random sequence generation, allocation concealment, blinding of participants and personnel, blinding of outcome assessment, incomplete outcome data, selective reporting, and other sources of bias. Disagreements were resolved through consensus.

### 2.4. Measures and Quantitative Synthesis—Meta-Analysis

Statistical analyses were performed using Stata version 18.0 (StataCorp, College Station, TX, USA). Continuous outcomes measured on the same scale (e.g., NIH-CPSI total and domain scores) were pooled as weighted mean differences (WMDs) with 95% confidence intervals (CIs), using final follow-up values at the time point closest to 12 weeks. Statistical heterogeneity was assessed using Cochran’s Q test and the I^2^ statistic. Given anticipated clinical heterogeneity related to shock wave generators, treatment protocols, and comparator regimens, a random-effects model was applied for the primary meta-analyses regardless of the level of statistical heterogeneity.

Prespecified subgroup analyses were conducted according to shock wave modality (focused, radial, or multifocal) and therapy strategy (monotherapy vs. add-on to standard medical therapy [SMT]). Sensitivity analyses included (1) leave-one-out analyses by sequentially excluding individual studies, and (2) a follow-up–restricted analysis including only trials with follow-up ≥12 weeks. The same effect measure (WMD) and random-effects model were applied. Publication bias was assessed by visual inspection of funnel plots when applicable; formal tests for small-study effects were not performed because fewer than 10 studies were included.

### 2.5. Certainty of Evidence Assessment

The certainty of the body of evidence for the primary outcome (total NIH-CPSI score at the follow-up time point closest to 12 weeks) was assessed using the Grading of Recommendations Assessment, Development and Evaluation (GRADE) framework. Two reviewers (Wu and Chen) independently rated the certainty of evidence as high, moderate, low, or very low. The assessment considered five domains: risk of bias (based on the Cochrane risk-of-bias tool for randomized trials, RoB 1.0), inconsistency, indirectness, imprecision, and publication bias, with discrepancies resolved by consensus. Because heterogeneity was substantial in the overall pooled analysis and modality-specific effects were prespecified, the certainty of evidence was assessed separately for each wave-generator modality subgroup (focused, radial, and multifocal). Publication bias was considered using funnel plot inspection when applicable; formal tests for small-study effects were not performed because fewer than 10 studies were included.

### 2.6. Use of Artificial Intelligence Tools

Generative AI tools, including ChatGPT version 5.2 (OpenAI) and Gemini 3 Pro (Google), were used during manuscript preparation solely to assist with English-language editing, improving textual clarity and refining figure presentation (e.g., layout and labels). These tools were not used to generate, extract, or modify study data and were not involved in study selection, risk-of-bias judgments, or statistical analyses. All AI-assisted revisions were reviewed and verified by the authors to ensure accuracy and consistency. ChatGPT version 5.2 and Gemini 3 Pro are web-based tools; therefore, a fixed software version number does not apply. Both were accessed on 24 January 2026.

## 3. Results

### 3.1. Study Selection

**Figure 1:**
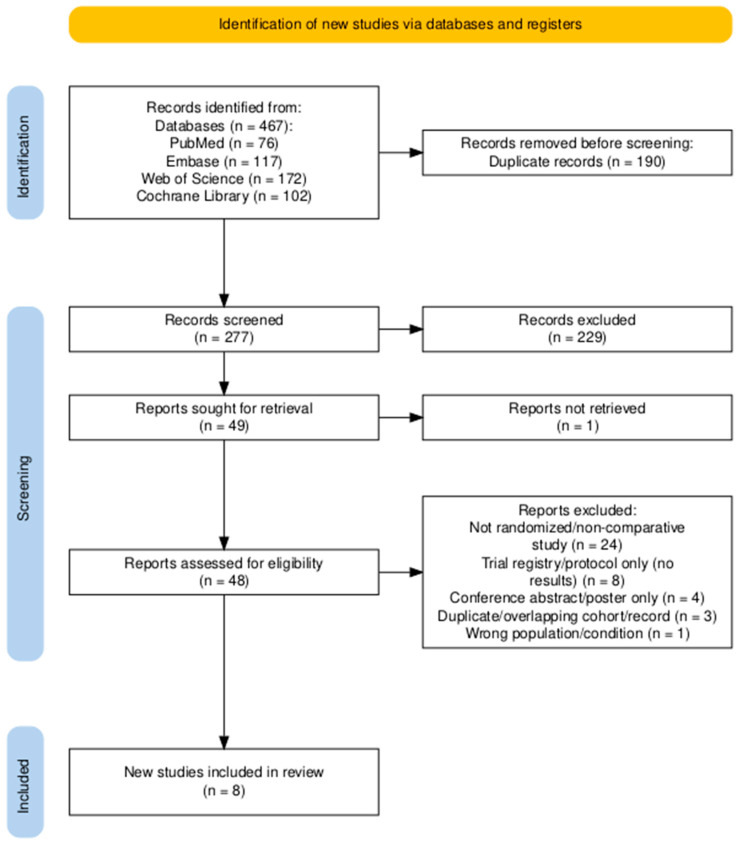
Preferred Reporting Items for Systematic Reviews and Meta-Analyses (PRISMA). Flow diagram [16].

The study selection process is summarized in the PRISMA flow diagram (Figure 1). The database search (January 2015 to 31 October 2025) identified 467 records (PubMed, n = 76; Embase, n = 117; Web of Science, n = 172; Cochrane Library, n = 102). After removal of 190 duplicate records, 277 records were screened by title and abstract, and 229 records were excluded. Forty-nine reports were sought for retrieval; one report could not be retrieved. Consequently, 48 full-text reports were assessed for eligibility, and 40 were excluded (not randomized/non-comparative study, n = 24; trial registry/protocol only without results, n = 8; conference abstract/poster only, n = 4; duplicate/overlapping cohort/record, n = 3; wrong population/condition, n = 1). Eight RCTs were included in the final qualitative and quantitative synthesis.

### 3.2. Characteristics of Included Studies and Risk of Bias Assessment

The analysis included eight RCTs involving a total of 455 participants (Table 1). All trials used a parallel-group design. Five trials were monotherapy (Li-ESWT vs sham), and three trials were add-ons to SMT (Li-ESWT + SMT vs. SMT) (Table 1). Treatment protocols varied; among trials reporting energy parameters, energy flux density (EFD) ranged from 0.096–0.26 mJ/mm^2^, and the number of sessions ranged from 4 to 12. Three wave-generator types were represented: focused (k = 4), radial (k = 2), and multifocal (k = 2). Multifocal devices were classified according to trial authors’ descriptions of generators delivering shock waves across multiple focal points/areas. Follow-up time points ranged from 4 to 48 weeks; for quantitative synthesis, outcomes were extracted using final follow-up values at the time point closest to the prespecified primary endpoint (12 weeks).

**Figure 2:**
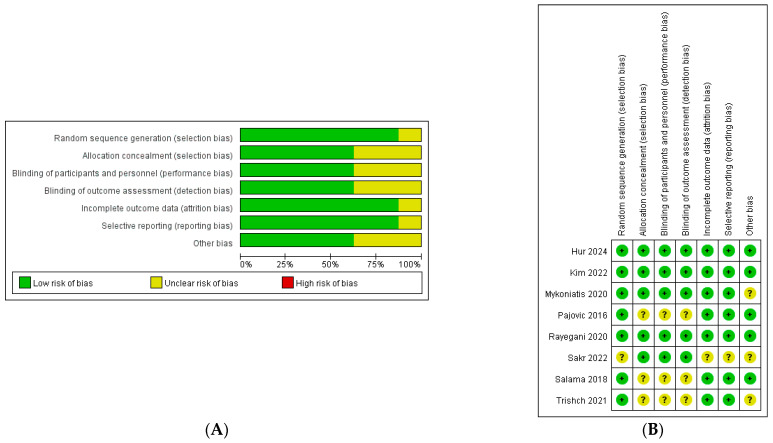
Risk-of-bias assessment of the included randomized controlled trials: (**A**) domain-level risk-of-bias summary for each individual study; (**B**) proportion of studies rated as having low, unclear, or high risk of bias in each domain. Symbols: “+” indicates low risk of bias; “?” indicates unclear risk of bias; “−” indicates high risk of bias [12,17,18,19,20,21,22,23].

The risk of bias (RoB 1.0) is summarized in Figure 2. Overall, the methodological quality was judged as moderate; while specific limitations were noted, no study received consistently high risk-of-bias judgments across domains. Random sequence generation was adequately described in most trials (7/8 low risk). In contrast, allocation concealment was frequently judged as unclear (3/8), largely due to insufficient reporting (e.g., Pajovic 2016 [12], Salama 2018 [23], and Trishch 2021 [19]). Similarly, blinding of participants/personnel and blinding of outcome assessment were rated as unclear in these three device-based trials, reflecting limited reporting and the practical challenges of blinding device interventions. Incomplete outcome data and selective reporting were generally low risk (7/8 each), with one trial judged as unclear for each domain. Other sources of bias were judged as unclear in three trials. Risk-of-bias considerations were incorporated into interpretation, and sensitivity analyses were performed to evaluate the robustness of pooled estimates.

### 3.3. Efficacy Outcomes

#### 3.3.1. Total NIH-CPSI Score

Baseline total NIH-CPSI scores were similar between groups. The pooled baseline comparison showed no significant difference between the Li-ESWT and control arms (WMD +0.56 points; 95% CI −0.41 to +1.53; *p* = 0.259), with negligible heterogeneity (I^2^ = 0.0%; Figure S1), suggesting minimal baseline imbalance.

**Figure 3:**
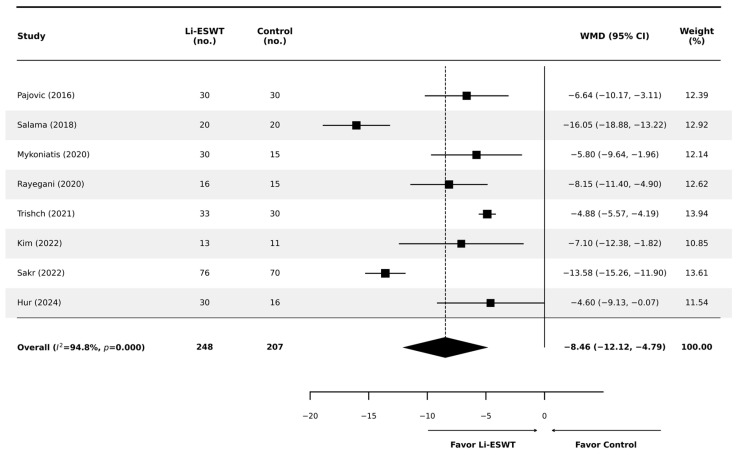
Forest plot comparing the effects of Li-ESWT versus control on total NIH-CPSI scores at 12 weeks. Squares represent individual study effect estimates (with size proportional to study weight), and diamonds represent pooled effect estimates (with width indicating the 95% confidence interval) [12,17,18,19,20,21,22,23].

At the 12-week primary endpoint (or the closest reported follow-up), Li-ESWT was associated with a significant improvement in total NIH-CPSI compared with controls (random-effects WMD −8.46 points; 95% CI −12.12 to −4.79; *p* < 0.001; Figure 3). The magnitude of benefit exceeded the commonly cited minimal clinically important difference (MCID) threshold (approximately 4–6 points), supporting clinical relevance. However, substantial heterogeneity was observed (I^2^ = 94.8%; *p* < 0.001), prompting prespecified subgroup analyses to explore potential effect modifiers.

**Figure 4:**
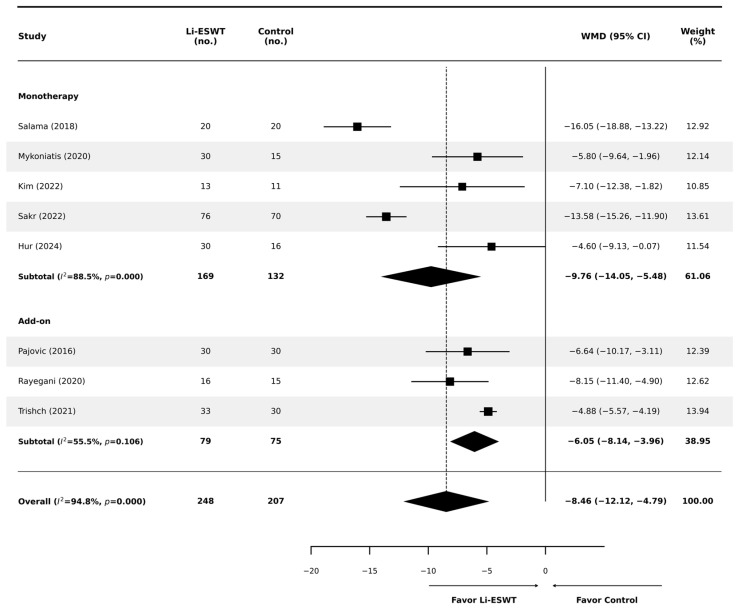
Subgroup analysis of total NIH-CPSI scores stratified by therapy strategy: monotherapy vs. add-on to SMT. Plot symbols are as in Figure 3 (squares = individual study estimates; diamonds = pooled estimates; diamond width = 95% CI) [12,17,18,19,20,21,22,23].

When stratified by therapy strategy (Figure 4), Li-ESWT remained superior in both trial designs. In monotherapy trials (k = 5), Li-ESWT showed a significant benefit (WMD −9.76; 95% CI −14.05 to −5.48; *p* < 0.001), although heterogeneity remained high (I^2^ = 88.5%). In add-on SMT trials where Li-ESWT was added to standard medical therapy (k = 3), Li-ESWT provided a significant add-on benefit (WMD −6.05; 95% CI −8.14 to −3.96; *p* < 0.001) with moderate heterogeneity (I^2^ = 55.5%). The test for subgroup differences was significant (*p* < 0.001); nevertheless, residual heterogeneity within the monotherapy subgroup suggests that therapy strategy alone does not fully account for between-study variability.

**Figure 5:**
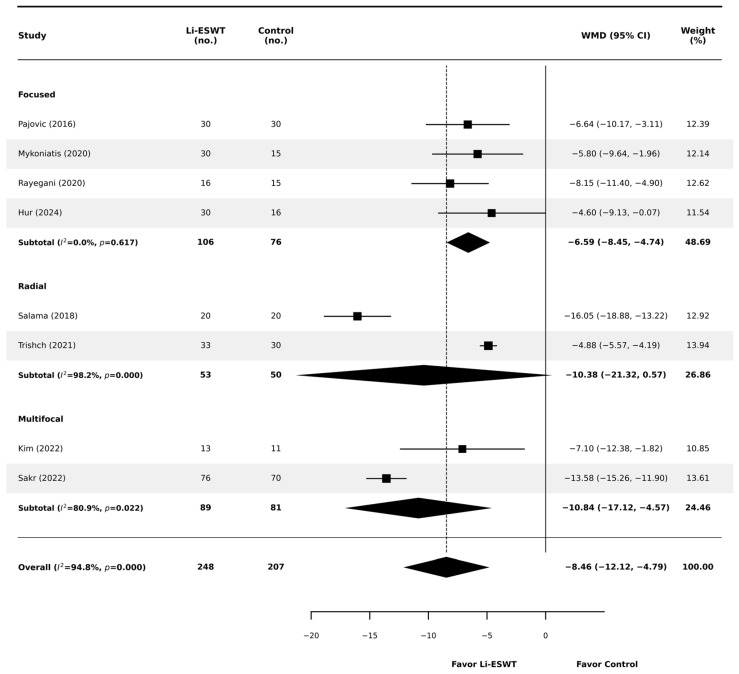
Subgroup analysis of total NIH-CPSI scores stratified by shockwave generator modality (focused, radial, and multifocal). Plot symbols are as in Figure 3 (squares = individual study estimates; diamonds = pooled estimates; diamond width = 95% CI) [12,17,18,19,20,21,22,23].

In contrast, stratification by wave-generator modality (Figure 5) showed marked differences in the pattern of effects and within-subgroup heterogeneity. Focused devices (k = 4) demonstrated a significant and homogeneous effect (WMD −6.59; 95% CI −8.45 to −4.74; *p* < 0.001; I^2^ = 0.0%). Radial devices (k = 2) showed a larger point estimate (WMD −10.38) but with extreme heterogeneity (I^2^ = 98.2%); the pooled estimate was imprecise and did not reach statistical significance (95% CI −21.33 to +0.57; *p* = 0.063). Multifocal devices (k = 2) also demonstrated a significant benefit (WMD −10.84; 95% CI −17.12 to −4.57; *p* = 0.001) but retained substantial heterogeneity (I^2^ = 81.0%). Given the small number of trials per subgroup and the absence of head-to-head randomized comparisons, modality-specific findings should be interpreted as exploratory and hypothesis-generating.

#### 3.3.2. Secondary Outcomes: Pain and Urinary Domains

Outcomes for NIH-CPSI domain scores were extracted at the prespecified primary follow-up (12 weeks) or, when 12-week data were unavailable, at the closest reported time point to 12 weeks to avoid double-counting within individual trials. For the pain domain, Li-ESWT was associated with a significant improvement compared with control (random-effects WMD −4.03 points; 95% CI −5.71 to −2.35; *p* < 0.05) (Figure S2), although heterogeneity remained substantial (I^2^ = 87.7%). The magnitude of pain reduction suggests that analgesic benefit is a major contributor to the overall improvement in total NIH-CPSI observed with Li-ESWT.

In contrast, the pooled effect on the urinary domain was small and not statistically significant (random-effects WMD −0.20 points; 95% CI −1.22 to 0.83; *p* = 0.706) (Figure S3), with considerable heterogeneity (I^2^ = 93.4%). This variability may reflect differences in baseline urinary symptom burden, concomitant pharmacotherapy (add-on designs), and follow-up timing across trials. Collectively, these findings indicate that the symptomatic benefit of Li-ESWT in CP/CPPS appears more consistent for pain relief than for urinary symptoms, and domain-specific effects should be interpreted alongside between-study heterogeneity.

### 3.4. Sensitivity Analyses and Publication Bias

Sensitivity analyses did not materially change the primary findings. A leave-one-out analysis was performed by sequentially omitting one study at a time. The pooled WMD for total NIH-CPSI remained statistically significant across all iterations, ranging from −7.35 (excluding Salama 2018) to −8.96 (excluding Hur 2024) (Figure S4), suggesting that the overall pooled effect was not driven by any single study. In an additional follow-up–restricted sensitivity analysis including only trials reporting ≥12-week outcomes (k = 5), the pooled effect remained significant (WMD −7.86; 95% CI −12.17 to −3.55), with substantial heterogeneity persisting (I^2^ = 95.5%) (Figure S5).

**Figure 6:**
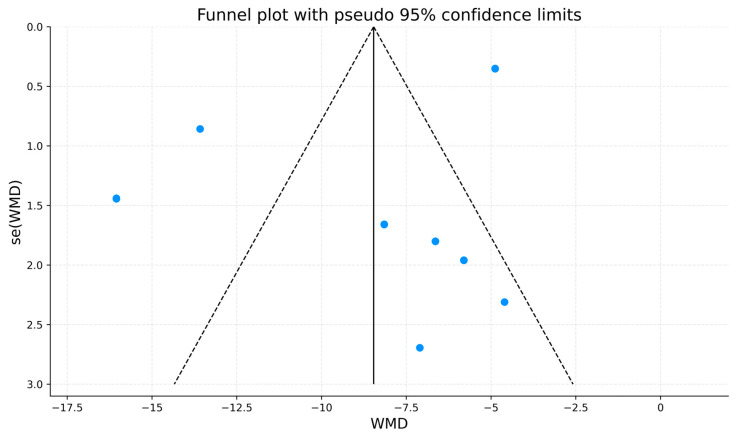
Funnel plot for the assessment of potential publication bias in studies reporting total NIH-CPSI scores. Blue dots represent individual studies; the solid vertical line indicates the pooled effect estimate; dashed lines indicate pseudo 95% confidence limits.

A funnel plot for the total NIH-CPSI outcome is presented for descriptive purposes (Figure 6). Visual inspection suggested possible asymmetry, with fewer small studies showing null or minimal effects. However, interpretation is constrained by the limited number of included studies (k = 8) and substantial heterogeneity (I^2^ = 94.8%). In line with methodological recommendations, formal statistical tests for small-study effects (e.g., Egger’s test) were not performed because they are underpowered when fewer than 10 studies are available. While publication bias cannot be excluded, any apparent asymmetry may also reflect clinical and methodological heterogeneity across trials, including differences in wave-generator modality, treatment protocols, comparator type, and follow-up timing, rather than selective reporting alone.

### 3.5. Certainty of Evidence (GRADE Assessment)

The certainty of evidence for the primary outcome (total NIH-CPSI) was assessed using the GRADE approach (Supplementary Table S2). Overall certainty for the pooled effect was rated as low, primarily due to very serious inconsistency (substantial between-study heterogeneity), with additional concerns regarding risk of bias commonly encountered in device-based trials.

In exploratory subgroup analyses by wave-generator modality, the certainty of within-modality effect estimates (Li-ESWT vs. control) was rated as moderate for focused devices, low for multifocal devices, and very low for radial devices, driven mainly by inconsistency and imprecision (only two trials and confidence intervals including no effect). Importantly, because no head-to-head randomized trials comparing device modalities were identified, any inference regarding differential efficacy between modalities is indirect and should be interpreted as hypothesis-generating. Moreover, none of the included RCTs stratified patients by CP/CPPS phenotype or presumed pain generator; therefore, depth-based considerations should be viewed as a conceptual framework for future trials rather than a practice recommendation.

## 4. Discussion

The management of chronic prostatitis/chronic pelvic pain syndrome (CP/CPPS) remains challenging. Although alpha-blockers, antibiotics, and anti-inflammatory agents are widely used, many patients experience only partial or short-lived relief, with persistent impairment in quality of life [5,24]. This likely reflects the multifactorial nature of CP/CPPS, involving pelvic floor dysfunction, altered microcirculation, and neurogenic inflammation [2,13,25]. Low-intensity extracorporeal shock wave therapy (Li-ESWT) has therefore been studied as a nonpharmacologic option with proposed effects on angiogenesis, pain signaling, and tissue repair [8,26]. However, results across trials have been inconsistent. This makes it important to consider technical factors, especially wave-generator modality, and differences in concomitant therapies.

To address heterogeneity noted in prior reviews [13,24], we stratified trials by shockwave modality. Focused devices showed consistent effects with negligible heterogeneity (I^2^ = 0.0%) and a pooled reduction of 6.59 points in total NIH-CPSI (95% CI −8.45 to −4.74). In contrast, the radial subgroup was based on only two trials with discordant results, leading to extreme heterogeneity (I^2^ = 98.2%) and an imprecise pooled estimate that did not reach statistical significance (*p* = 0.063). Multifocal devices showed a significant pooled effect but remained heterogeneous (I^2^ = 80.9%). These patterns are compatible with known physical differences between generators [13]. Focused systems create a focal zone with deeper tissue penetration, which may better reach the prostate and pelvic floor musculature [27,28]. Radial devices disperse energy more superficially because of ballistic generation [27,29]. As a result, outcomes with radial devices may be more variable across settings and more sensitive to patient anatomy and applicator positioning. Importantly, because no head-to-head randomized trials comparing modalities were identified, these modality-specific estimates should be interpreted as exploratory rather than definitive. Moreover, none of the included RCTs stratified patients by CP/CPPS phenotype or presumed pain generator; therefore, depth-based considerations should be viewed as a conceptual framework for future trials rather than a practice recommendation. Treating all generators as interchangeable may therefore obscure clinically relevant differences, and prior comparative work in other urologic applications of shockwave therapy also suggests that treatment response can vary by generator type [28].

Our domain-specific results also help position Li-ESWT clinically. The overall benefit was driven mainly by improvement in the pain domain (WMD −4.01), consistent with proposed neuromodulatory and anti-inflammatory mechanisms [25,26]. In contrast, no significant benefit was observed in the urinary domain (WMD −0.20; *p* = 0.706). This suggests that Li-ESWT may act primarily as a pain-modulating intervention rather than directly improving voiding symptoms.

Li-ESWT also appeared beneficial in both monotherapy and add-on SMT trials. In subgroup analyses, benefits were observed for both monotherapy (k = 5; WMD −9.76; I^2^ = 88.5%) and add-on SMT trials (k = 3; WMD −6.05; I^2^ = 55.5%) with a significant subgroup difference test (*p* < 0.001). This is consistent with the idea of multimodal management in complex pelvic pain syndromes [24]. Similar tiered strategies have been explored in related prostatitis conditions, such as adding nutraceuticals to antibiotics in chronic bacterial prostatitis [6]. In CP/CPPS, Li-ESWT may address neuromuscular and local inflammatory components within the UPOINT framework, while medications target other domains [12,24]. Indeed, empirical evidence supports this role; Guu et al. (2018) documented significant clinical salvage in patients refractory to the conventional “3-As” regimen [30], while Wu et al. (2021) reported enhanced pain alleviation and functional outcomes in similarly treatment-resistant cohorts [31]. Taken together, these findings support Li-ESWT as a reasonable nonpharmacologic adjunct. However, heterogeneity persisted—particularly in the monotherapy subgroup—likely reflecting differences in co-interventions and protocols. Therefore, the subgroup findings should not be used to infer that one treatment strategy is superior to another.

Regarding safety, Li-ESWT appeared well tolerated in the included RCTs. Among trials reporting safety outcomes, no serious treatment-related adverse events were described, and reported events were infrequent, mild, and self-limiting. Rayegani et al. reported transient hematuria and hematospermia in a small subset of patients, and Hur et al. reported one case of a first-degree skin burn that resolved within one week [18,22]. In combination trials, systemic adverse effects were reported but were more likely related to concomitant pharmacotherapy than to shockwave application. Nevertheless, safety reporting was not uniform across studies, and available data are limited to short-to-medium follow-up.

Several limitations should be noted. Only eight RCTs were included, limiting the reliability of publication bias assessment; funnel plot asymmetry should be interpreted cautiously given < 10 studies and substantial heterogeneity. Protocols varied across trials (energy settings and schedules), and blinding is inherently difficult in device-based interventions, raising concerns about performance bias. Follow-up ranged from 4 to 48 weeks, and long-term durability remains uncertain. Future studies should use standardized protocols and directly compare device modalities to clarify whether generator type modifies treatment response. A follow-up–restricted sensitivity analysis including only trials reporting ≥12-week outcomes remained significant, although substantial heterogeneity persisted, suggesting that follow-up timing is not the sole driver of between-study variability.

## 5. Conclusions

In conclusion, Li-ESWT offers clinically meaningful symptom relief for CP/CPPS, primarily driven by pain reduction rather than improvements in urinary function. Exploratory analyses suggest that treatment effects may vary by generator type: focused devices demonstrated consistent therapeutic benefits, whereas radial devices exhibited marked heterogeneity with imprecise, non-definitive estimates; multifocal devices showed significant benefit but remained heterogeneous. While these patterns are compatible with physical differences in energy delivery and tissue penetration, they remain hypothesis-generating in the absence of direct head-to-head comparisons. Clinically, Li-ESWT should be positioned not as a stand-alone definitive therapy but as an integral component of a multimodal management strategy. Future research should prioritize standardized, adequately powered head-to-head comparative trials to determine whether generator-specific characteristics translate into differential clinical response.

## Figures and Tables

**Table 1:** Characteristics and treatment parameters of the randomized controlled trials included in the meta-analysis.

Author, Year	Study Design	Intervention(n)	Control(n)	EFD (mJ/mm^2^)	Sessions	Device Type	Follow-Up (Weeks)	Outcome
Mykoniatis 2020 [17]	RCT	Li-ESWT (30)	Sham (15)	0.096	6	Focused	4, 12, 24	NIH-CPSI, VAS, IPSS, IIEF
Pajovic 2016 [12]	RCT	Li-ESWT + SMT (30)	SMT (30)	0.25	12	Focused	12, 24	NIH-CPSI, Qmax
Rayegani 2020 [18]	RCT	Li-ESWT + SMT (16)	SMT (15)	0.25	4	Focused	4, 12	NIH-CPSI, VAS, IPSS, Qmax, IIEF
Trishch 2021 [19]	RCT	Li-ESWT + SMT (33)	SMT (30)	Not specified	8	Radial	12, 24	NIH-CPSI
Kim 2022 [20]	RCT	Li-ESWT (13)	Sham (11)	0.26	8	Multifocal	4	NIH-CPSI, IPSS, IIEF-5, VAS
Sakr 2022 [21]	RCT	Li-ESWT (76)	Sham (70)	0.25	4	Multifocal	4, 12, 24, 48	NIH-CPSI, IPSS, VAS, IIEF-5
Hur 2024 [22]	RCT	Li-ESWT (30)	Sham (16)	0.25	8	Focused	4	NIH-CPSI, IIEF-EF
Salama 2018 [23]	RCT	Li-ESWT (20)	Sham (20)	Not specified	8	Radial	8	NIH-CPSI

Note: SMT = standard medical therapy; monotherapy trials compared Li-ESWT vs. sham, whereas add-on trials compared Li-ESWT + SMT vs. SMT alone. EFD = energy flux density (mJ/mm^2^); values are reported as described in each trial and “Not specified” indicates the parameter was not reported. Sessions indicate the total number of Li-ESWT treatment sessions as reported in each trial. Device type (focused/radial/multifocal) was classified according to trial reports/manufacturer descriptions; “multifocal” refers to generators delivering shock waves across multiple focal points/areas or a broadened focal field compared with conventional unifocal focused systems. Follow-up (weeks) lists all follow-up time points reported in each trial; for quantitative synthesis, one time point per study was used (final follow-up value closest to 12 weeks). Abbreviations (outcomes): NIH-CPSI, National Institutes of Health Chronic Prostatitis Symptom Index; VAS, visual analog scale; IPSS, International Prostate Symptom Score; IIEF, International Index of Erectile Function; Qmax, maximum urinary flow rate.

## Data Availability

The data supporting the findings of this study were obtained from previously published clinical trials cited in the article. The extracted datasets used for the meta-analysis are available from the corresponding author upon reasonable request.

## Supplementary Materials

The following supporting information can be downloaded at: https://www.mdpi.com/article/10.3390/jcm15031270/s1. Figure S1: Forest plot of baseline total NIH-CPSI scores; Figure S2: Forest plot of NIH-CPSI pain domain scores; Figure S3: Forest plot of NIH-CPSI urinary domain scores; Figure S4: Sensitivity analysis (leave-one-out) for total NIH-CPSI scores; Figure S5: Sensitivity analysis (forest plot) of trials with follow-up ≥12 weeks; Table S1: Database-specific search strategies; Table S2: GRADE Evidence Profile (Summary of Findings); Table S3: PRISMA 2020 Checklist.

## Abbreviations

The following abbreviations are used in this manuscript:

CP/CPPS	Chronic prostatitis/chronic pelvic pain syndrome
Li-ESWT	Low-intensity extracorporeal shock wave therapy
ESWT	Extracorporeal shock wave therapy
NIH-CPSI	National Institutes of Health Chronic Prostatitis Symptom Index
VAS	Visual analogue scale
IPSS	International Prostate Symptom Score
IIEF	International Index of Erectile Function
EFD	Energy flux density
3-As	Combined regimen of antibiotics, alpha-blockers, and anti-inflammatory agents
