# Supplementary material for: Optimizing the Use of Extracorporeal Shock Wave Therapy for CP/CPPS: A Modality-Based Systematic Review and Meta-Analysis Comparing Focused and Radial Devices
Source: J Clin Med. 2026 Feb 5;15(3):1270. doi: 10.3390/jcm15031270 (PMC12897672; doi:10.3390/jcm15031270)
Supplement: Supplementary file 1 [file jcm-15-01270-s001.zip › Supplementary_Table_S1.pdf]

Supplementary Table S1. Database-specific search strategies

Database	Platform / Interface	Date searched (YYYY-MM-DD)	Date limits applied	Other limits / filters	Full search strategy (as run)	Records retrieved (n)
PubMed	pubmed.ncbi.nlm.nih.gov	2026/01/17	2015/01/01–2025/10/31	None	(("Prostatitis"[Mesh] OR prostatitis[tiab] OR "chronic prostatitis"[tiab] OR "chronic pelvic pain syndrome"[tiab] OR CP/CPPS[tiab] OR "pelvic pain"[tiab] OR prostatodynia[tiab]) AND ("extracorporeal shock wave therapy"[tiab] OR "shock wave therapy"[tiab] OR shockwave[tiab] OR "shock wave"[tiab] OR ESWT[tiab] OR Li-ESWT[tiab] OR LiESWT[tiab]))	76
Embase	Elsevier Embase.com	2026/01/17	2015/01/01–2025/10/31	None (screening performed after export)	('chronic pelvic pain syndrome'/exp OR 'prostatitis'/exp) AND ('shock wave therapy'/exp OR 'extracorporeal shockwave therapy'/exp)	117
Web of Science Core Collection	Clarivate Web of Science	2026/01/17	2015–2025 (Timespan/Refine applied in interface)	Core Collection; no additional filters (screening performed after export)	TS=(prostatitis OR "chronic prostatitis" OR "chronic pelvic pain syndrome" OR CP/CPPS OR prostatodynia OR "pelvic pain") AND TS=("shock wave" OR shockwave OR ESWT OR "extracorporeal shock wave" OR "shock wave therapy" OR "extracorporeal shock wave therapy" OR Li-ESWT OR LiESWT)	172
Cochrane CENTRAL	Cochrane Library (CENTRAL)	2026/01/17	2015/01/01–2025/10/31 (filter applied in interface)	Trials register (CENTRAL); no additional RCT filter applied	(prostatitis OR "chronic prostatitis" OR "chronic pelvic pain syndrome" OR CP/CPPS OR prostatodynia OR "pelvic pain") AND ("shock wave" OR shockwave OR ESWT OR "extracorporeal shock wave" OR "shock wave therapy" OR "extracorporeal shock wave therapy" OR Li-ESWT OR LiESWT)	99
Cochrane SR	pubmed.ncbi.nlm.nih.gov	2026/01/17	2015/01/01–2025/10/31	None	(prostatitis OR "chronic prostatitis" OR "chronic pelvic pain syndrome" OR CP/CPPS OR prostatodynia OR "pelvic pain") AND ("shock wave" OR shockwave OR ESWT OR "extracorporeal shock wave" OR "shock wave therapy" OR "extracorporeal shock wave therapy" OR Li-ESWT OR LiESWT)	3

Footnote: No study-design filter (e.g., RCT filter) was applied at the search stage; eligibility was determined during title/abstract and full-text screening.Searches were executed on 17 Jan 2026 using a prespecified end-date filter (up to 31 Oct 2025)
