# Supplementary material for: Optimizing the Use of Extracorporeal Shock Wave Therapy for CP/CPPS: A Modality-Based Systematic Review and Meta-Analysis Comparing Focused and Radial Devices
Source: J Clin Med. 2026 Feb 5;15(3):1270. doi: 10.3390/jcm15031270 (PMC12897672; doi:10.3390/jcm15031270)
Supplement: Supplementary file 1 [file jcm-15-01270-s001.zip › Supplementary_Table_S2.pdf]

Supplementary Table S2. GRADE Evidence Profile (Summary of Findings)

Outcome & Subgroup	No. of Participants (Studies)	Certainty of Evidence (GRADE)	Absolute effect (MD, points) [95% CI]
Total NIH-CPSI (overall)	455(8 RCTs)	⊕⊕◯◯LOW[a]	−8.46 [−12.12 to −4.79]
Focused Li-ESWT	182(4 RCTs)	⊕⊕⊕◯MODERATE[b]	−6.59 [−8.45 to −4.74]
Radial Li-ESWT	103 (2 RCTs)	⊕◯◯◯VERY LOW[c]	−10.38 [−21.32 to 0.57]
Multifocal Li-ESWT	170 (2 RCTs)	⊕⊕◯◯LOW[d]	−10.84 [−17.12 to −4.57]

* Explanations:

a. Downgraded one level for risk of bias because blinding and/or allocation concealment were frequently unclear in device-based trials, reflecting limitations in reporting and the practical challenges of sham procedures.

b. Downgraded two levels for very serious inconsistency due to substantial heterogeneity in the pooled estimate (overall I^2= 94.8%), suggesting important between-study differences that could not be fully explained.

c. Downgraded two levels for very serious inconsistency and one level for serious imprecision in the radial subgroup, as only two trials were available with markedly different effect sizes (I^2= 98.2%) and the pooled confidence interval included no effect.

d. Downgraded one level for serious inconsistency and one level for serious imprecision in the multifocal subgroup, given the small number of trials (n = 2), substantial heterogeneity (I^2 = 80.9%), and wide confidence intervals.

Note: Modality-specific estimates represent within-modality comparisons versus control and are derived from indirect between-trial comparisons; no head-to-head randomized trials comparing modalities were available. Modality subgroup findings should be interpreted as exploratory/hypothesis-generating. Commonly used MCID for total NIH-CPSI is approximately 4–6 points.
